# Tagging to endogenous genes of *Plasmodium falciparum* using CRISPR/Cas9

**DOI:** 10.1186/s13071-017-2539-0

**Published:** 2017-12-02

**Authors:** Dexuan Kuang, Jichen Qiao, Zhou Li, Weiwei Wang, Hui Xia, Lubin Jiang, Jiejie Dai, Qiang Fang, Xueyu Dai

**Affiliations:** 1 Institute of Medical Biology, Chinese Academy of Medical Science and Peking Union Medical College, Kunming, Yunnan 650118 China; 2 Department of Microbiology and Parasitology, Chinese Academy of Medical Science and Peking Union Medical College, Kunming, Yunnan 650118 China; 3 Anhui Key Laboratory of Infection and Immunity at Bengbu Medical College, Bengbu, 233030 China; 4 Institut Pasteur of Shanghai, Chinese Academy of Sciences, Shanghai, 200031 China

**Keywords:** *Plasmodium falciparum*, CRISPR/Cas9, Gene editing, Gene tagging

## Abstract

**Background:**

*Plasmodium falciparum* is the deadliest malaria parasite. Currently, there are seldom commercial antibodies against *P. falciparum* proteins, which greatly limits the study on *Plasmodium*. CRISPR/Cas9 is an efficient genome editing method, which has been employed in various organisms. However, the use of this technique in *P. falciparum* is still limited to gene knockout, site-specific mutation and generation of green fluorescent protein (GFP) reporter line with disruption of inserted sites.

**Results:**

We have adapted the CRISPR/Cas9 system to add commercial tag sequences to endogenous genes of *P. falciparum.* To add HA or HA-TY1 tags to ck2β1, ck2α and stk, pL6cs-hDHFR-ck2β1/ck2α/stk was constructed, which contained sequences of tags, specific homologous arms, and sgRNA. The *P. falciparum* 3D7 strain was subsequently transfected with pUF1-BSD-Cas9 and pL6cs-hDHFR-ck2β1/ck2α/stk plasmids via electroporation. After that, BSD and WR99210 drugs were added to the culture to screen parasites containing both plasmids. Twenty days after electroporation, live parasites appeared and were collected to check the tagging by PCR, DNA sequencing, Western blotting and immuno-fluorescence assays. The results showed that the tags were successfully integrated into the C-terminus of these three proteins.

**Conclusions:**

We have improved the method to integrate tags to *Plasmodium falciparum* genes using the CRISPR/Cas9 method, which lays the foundation for further study of *Plasmodium falciparum* at the molecular level.

**Electronic supplementary material:**

The online version of this article (10.1186/s13071-017-2539-0) contains supplementary material, which is available to authorized users.

## Background

Malaria is an infectious disease which presents a serious threat to human life and health. *Plasmodium falciparum* is the deadliest malaria parasite, which gives rise to many clinical cases around the world each year [1]. Malaria can cause a variety of complications, such as anemia, hepatosplenomegaly, cerebral malaria, miscarriage and death [2–4]. Currently, there is still no effective vaccine to prevent malaria, so the strategy for controlling the disease is dependent on anti-malarial drugs [5–7]. The emergence of artemisinin has effectively controlled the spread of malaria. However, artemisinin-resistant parasites have appeared in Southeast Asia [8–10]. Thus, malaria prevention and therapy remain an important mission.

The study of *Plasmodium falciparum* at the molecular level can significantly promote the development of malaria vaccines and anti-malarial drugs [11, 12]. Before the emergence of CRISPR/Cas9, single or double-crossover recombination strategies were used to edit genes in *P. falciparum* [13–15]. Briefly, plasmids were used to transfect parasites and kept as episomes [14]. Then several on/off drug selection cycles were carried out to isolate parasites with the desired chromosomal integration event. This method was very inefficient and required months to achieve the desired gene modification [16]. The *Streptococcus pyogenes* CRISPR/Cas9 system has enhanced gene editing in various organisms [17–19]. Cas9 endonuclease is guided to a specific target DNA site by the single guide RNA (sgRNA) and subsequently induces double-strand breaks (DSBs) at this site. The DSBs are then repaired by homologous recombination using donor DNAs since the canonical nonhomologous end-joining (NHEJ) is deficient in *Plasmodium* [20]. This technique has already been used in *P. falciparum* for gene knock out, generating single-nucleotide substitutions and a green fluorescent protein (GFP) reporter line with disruption of inserted sites [17, 19, 21], but the adaption of this system for adding tags to *P. falciparum* genes has not been reported yet.

Currently, there are few commercially available antibodies against *P. falciparum* proteins, which greatly limits the study of *Plasmodium*. Based on CRISPR/Cas9, we adapted this system to add commercial tag sequences (such as HA, TY1) to *P. falciparum* genes. We show that this tagging strategy is comparatively quick, as it only takes 20 days to achieve transgenic *P. falciparum*, and efficient since we successfully obtained three distinct tagged genes in three trials. This tagging system lays the foundation for molecular studies on *Plasmodium falciparum*.

## Methods

### Plasmid construction and preparation

Based on pUF1-Cas9 and pL6cs plasmids, kindly provided by Jose-Juan Lopez-Rubio, pUF1-BSD-Cas9 and pL6cs-hDHFR-ck2β1/ck2α/stk plasmids were constructed to create our tagging system.

pUF1-BSD-Cas9 expresses Cas9 endonuclease and blasticidin S deaminase (BSD). The pUF1-Cas9 vector was digested with *BamH*I and *Hind*III to remove the yDHODH coding sequences. The BSD coding sequences from pCC4 [22] were amplified by PCR and then inserted in the linearized pUF1-Cas9 using in-fusion cloning kit (Vazyme Biotech, Nanjing, China).

The pL6cs-hDHFR-ck2β1/ck2α/stk plasmids, which offer donor DNAs and sgRNAs, were constructed in multiple steps. First, the pL6cs plasmid was digested with *Pvu*II and *Asc*I, and the yFCU expression cassette was replaced with the annealed DNA fragment to introduce some new restriction site cassettes (Additional file 1) using in-fusion system to generate pL6cs-hDHFR vector. Next, the left and right homologous arms (i.e. donor DNAs) were amplified separately by PCR from genomic DNA of *P. falciparum* 3D7 (primers P3/P4 and P5/P6 for ck2β1, P15/P16 and P17/P18 for ck2α, P25/P26 and P27/P28 for stk).

For pL6cs-hDHFR-ck2β1, the linker-HA motif was amplified from synthesized HA DNA using P7/P8, producing a soft linker, HA and overlapped sequences to ck2β1 homologous arms. Similarly, for pL6cs-hDHFR-ck2α and pL6cs-hDHFR-stk plasmids, the linker-HA-TY1 motif was amplified from synthesized HA-TY1 DNA using P19/P20 or P29/P30, producing a soft linker, HA-TY1 and overlapped sequences to ck2α or stk homologous arms.

Then, the 1st bridging PCR was run from linker-HA or linker-HA-TY1 motif mixed with respective left homologous arm (P3/P8 for ck2β1, P15/P20 for ck2α, and P27/P30 for stk). The 2nd bridging PCR was run using the first bridging PCR product mixed with right homologous arm, respectively (P9/P10 for ck2β1, P21/P22 for ck2α and P26/P27 for stk). To prevent the already edited genomic DNA from being recognized and cut again by Cas9 after successful tagging, DNA sequences which expressed sgRNAs and PAM motif in donor DNAs (i.e. homologous arms) were mismatched to synonymous mutations. For ck2α and stk, the NGG was close to the stop codon and thus the mismatched mutations were introduced during the PCRs of left homologous arms from genomic DNA. Subsequently, the second bridging PCR products for ck2α and stk were inserted into the pL6cs-hDHFR vector which was linearized with *Asc*I and *Afl*II using an in-fusion cloning kit to create transitional-pL6cs-hDHFR-ck2β1 and transitional-pL6cs-hDHFR-stk plasmids.

For ck2β1, a mismatched mutation was introduced by extra PCRs using the 2nd bridging PCR product and two pairs of primers separately (P9/P12 and P11/P10). These two PCR products were mixed as template for final PCR using P9/P10 primers. This final DNA fragment was inserted into pL6cs-hDHFR vector linearized with *Eco*RI and *Nco*I using the in-fusion system to generate a transitional-pL6cs-hDHFR-ck2β1 plasmid.

The constructs of transitional-pL6cs-hDHFR-ck2β1/ck2α/stk were transformed into competent cells of XL-10. The plasmids were then extracted using Plasmid Mini Kit (Qiagen, Dusseldorf, Germany) and checked with restriction enzyme digestion as well as DNA sequencing. After the correct transitional-pL6cs-hDHFR-ck2β1/ck2α/stk plasmids were obtained, these transitional plasmids were linearized with *Avr*II & *Xho*I. The primers P13/P14, P23/P24 and P31/P32 were used to synthesize the DNA sequences expressing sgRNAs of ck2β1, ck2α and stk, respectively. After being annealed, DNA fragments of P13/P14, P23/P24, P31/P32 were respectively inserted into linearized transitional-pL6cs-hDHFR-ck2β1/ck2α/stk plasmids using in-fusion kit. The constructs were transformed into competent cells of XL-10 again and then extracted with Plasmid Mini Prep Kit. These final plasmids were confirmed by restriction enzyme digestion and DNA sequencing. The confirmed plasmids were isolated with Plasmid Mega Kit (Qiagen) and further used for electroporation to generate *P. falciparum* transgenic strains.

### *Plasmodium* transfection with plasmids

Cytomix buffer was made according to the previous report and kept at −20 °C [23]. *Plasmodium* 3D7 was cultured in fresh human red blood cells at 37 °C, 5% CO_2_, 5% O_2_ in RPMI-1640 medium containing 5 g/l Albumax. Parasites were synchronized with Percoll [24]. Fifteen hours after Percoll, the parasites had developed to the ring stage with around 5% parasitemia. The iRBCs (infected red blood cells) containing parasites were washed twice with 1× cytomix just before electroporation. The electroporation mixture contained 50 μg of pUF1-BSD-Cas9 plasmid (25 μl), 50 μg of pL6cs-hDHFR-ck2α (25 μl), 100 μl iRBC, 150 μl 2× Cytomix, and 100 μl H_2_O. The same mix formula was used for ck2β1 and stk. Parameters during electroporation performed with GenePulser Xcell (Bio-Rad, Hercules, California, USA) were set as follows: voltage is 310 V, capacity is 950 μF, electric resistance is infinite; cuvette gap is 2 mm. After electroporation, the parasites were transferred into flasks with medium, and cultured in the incubator with 5% O_2_, 5% CO_2_ at 37 °C. One day after electroporation, the iRBCs were smeared on slides and stained with Giemsa solution; usually, the parasitemia was around 2.5% at this time. BSD (selection marker encoded by pUF1-BSD-Cas9 plasmid) and WR99210 drugs (selection marker encoded by pL6cs-hDHFR-ck2β1/ck2α/stk plasmid) were freshly added to the culture every two days, to kill those parasites without episomal pUF1-BSD-Cas9 plasmid or pL6cs-hDHFR-ck2β1/ck2α/stk. All primer (from P1 to P36) and sgRNA sequences used for constructing plasmids can be seen in Additional file 1.

### Confirmation of tagging via PCR and DNA sequencing

Twenty days after electroporation, live *P. falciparum* appeared and were collected for genomic DNA isolation. A PCR check was carried out with specific primers (P1/P2, as indicated in Fig. 2) and the genomic DNAs as template. After PCR, if live *P. falciparum* were 100% tagged, the two drugs were not added into the culturing; if the live *P. falciparum* was NOT 100% tagged, both drugs were continuously added to the culture until the tagged population reached 100%. The PCR product from wild-type 3D7 genomic DNA was used as negative control of tagging. PCR products were analyzed on agarose gels, and products with the expected size were sent for sequencing to confirm the expected tagging.

### Confirmation of tagging via western blotting

Successfully transgenic parasites were cultured in flasks. When parasitemia passed 5%, iRBCs were collected and incubated with 0.15% saponin lysis solution on ice for 7 min. After centrifugation, the supernatant was removed. The *P. falciparum* pellet was resuspended in appropriate volume of cell lysate buffer and broken with sonication. After high-speed centrifugation at low temperature, the supernatant was placed in new tubes, mixed with SDS-PAGE loading buffer and denatured at 98 °C for 5 min. After cooling, the samples were loaded to SDS-PAGE and transferred to PVDF membrane. Integrated Tags were detected by using anti-TY1 antibody (Sigma-Aldrich, Saint Louis, USA) or anti-HA antibody (Abcam, Massachusetts, USA).

### Immunofluorescence assay (IFA)

IFA was performed as previously described [25]. Briefly, *P. falciparum* was synchronized with Percoll and then collected at ring, trophozoite or schizont stage. Infected red blood cells, were fixed with 4% paraformaldehyde for 20 min, and then washed 3 times with PBS, and permeabilized with 0.15% Trion X-100. Parasites were incubated overnight at 4 °C with mouse anti-TY1 antibody (Sigma-Aldrich, Saint Louis, USA) in PBS buffer containing 5% BSA (bovine serum albumin). After three washes with PBS, parasites were incubated with goat anti-mouse DyLight 550 antibody (Thermo Fisher Scientific, Waltham, USA) at 37 °C for 2 h and washed again 3 times with PBS. Parasites were placed on slides to form a monolayer, air-dried, and then sealed with mounting medium containing DAPI (VECTOR, California, USA). Pictures were taken under Leica S5 confocal microscope.

## Results

### Successful construct for three genes

ck2β1 (PF3D7_1103700), ck2α (PF3D7_1108400) and stk (PF3D7_0321400) genes, encode casein kinase 2 beta subunit 1, casein kinase 2 alpha subunit, and a putative protein kinase, respectively. Immunofluorescence assay (IFA), chromatin immunoprecipitation (ChIP) and co-immunoprecipitation (Co-IP) studies were required for these three proteins. However, there are no commercially available antibodies against these proteins. Thus, we decided to add commercial tag sequence to these three genes using CRISPR/Cas9 technology.

**Fig. 1:**
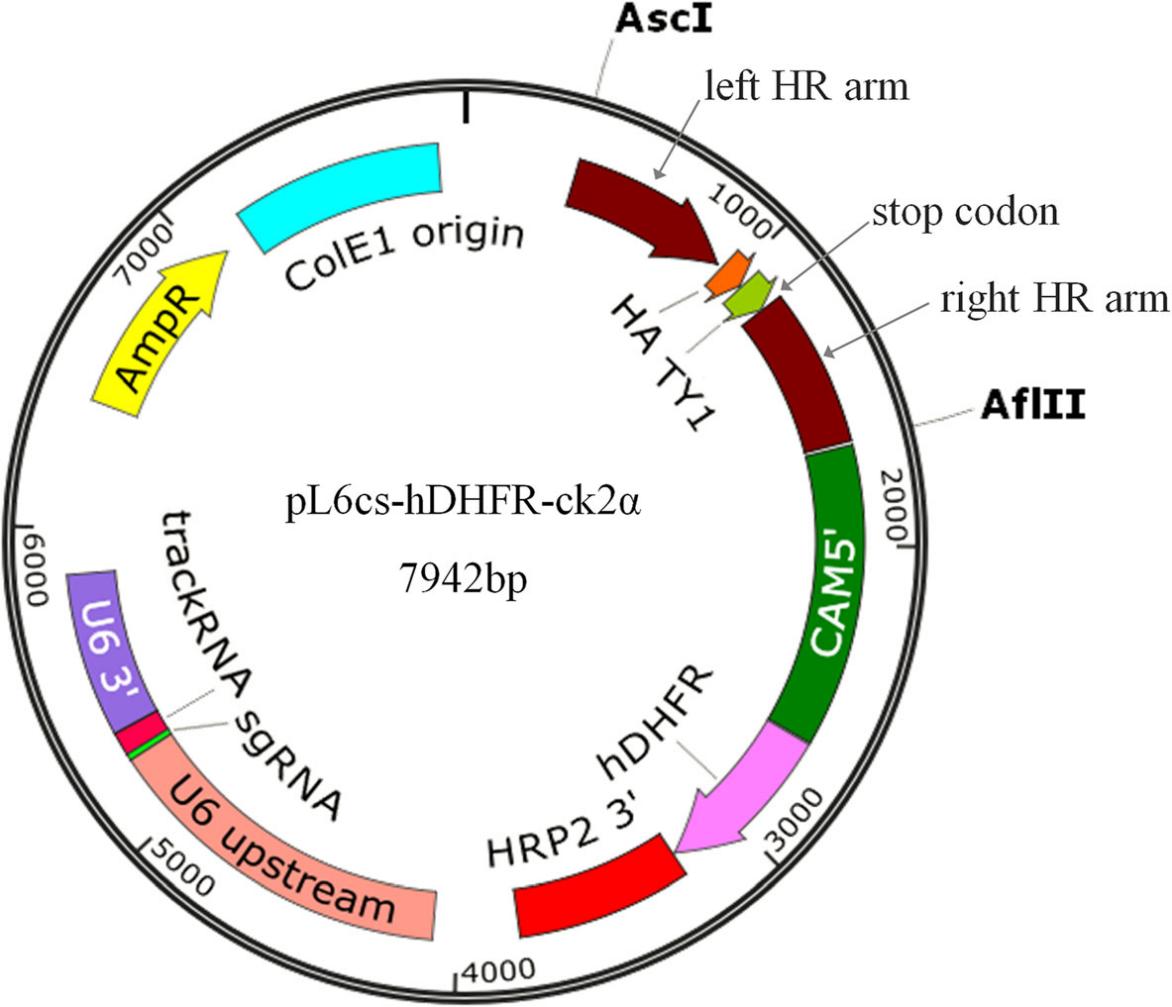
Plasmid map of pL6cs-hDHFR-ck2α. The HA-TY1 tag coding sequences were placed in the middle of the left and right homologous arms by the bridging PCR method. This continuous DNA fragment was then inserted into the pL6cs-hDHFR vector linearized with *Asc*I and *Afl*II by using an in-fusion method. The plasmid map of pL6cs-hDHFR-ck2α was used as a map example. The plasmid maps offering donor DNAs for ck2β1 and stk are similar to this ck2α map, except homologous arms and sgRNA expressing cassettes are gene specific

The pUF1-BSD-Cas9 plasmid, which offered Cas9 endonuclease and blasticidin S deaminase (BSD), was constructed from pUF1-Cas9 plasmid by changing drug selection marker from yDHODH into BSD. The plasmids which provide donor DNAs and sgRNA were constructed from the pL6cs plasmid after several modification steps. First, gene-specific DNA cassettes expressing sgRNAs were chosen from sequences around stop codon and inserted to pL6cs vector. Secondly, since tags were fused to the C-terminal ends of ck2β1, ck2α and stk, two ~550 bp genomic DNA sequences flanking the stop codon were chosen as homologous arms. Then, HA or HA-TY1 tag sequences were fused between the left and the right homologous arm sequences by bridge PCR. Each continuous sequence containing homologous arms and one of the above tags were inserted into pL6cs-hDHFR vector (Fig. 1). To prevent the already edited genomic DNA from being recognized and cut again by Cas9 after successful tagging, DNA sequences which expressed sgRNAs and PAM motif in donor DNAs (i.e. homologous arms) was mismatched to synonymous mutation.

All constructs were screened with enzyme digestion and DNA sequencing to ensure that the open reading frame for each gene was correct. After that, *P. falciparum* 3D7 strain was subsequently transfected with 50 μg pL6cs-hDHFR-ck2β1/ck2α/stk (donor DNA) and 50 μg pUF1-BSD-Cas9 plasmids via electroporation. To select the successful transfection of both plasmids, BSD and WR99210 were added to medium one day after electroporation.

### Successful tagging checked by PCR and DNA sequencing

**Fig. 2:**
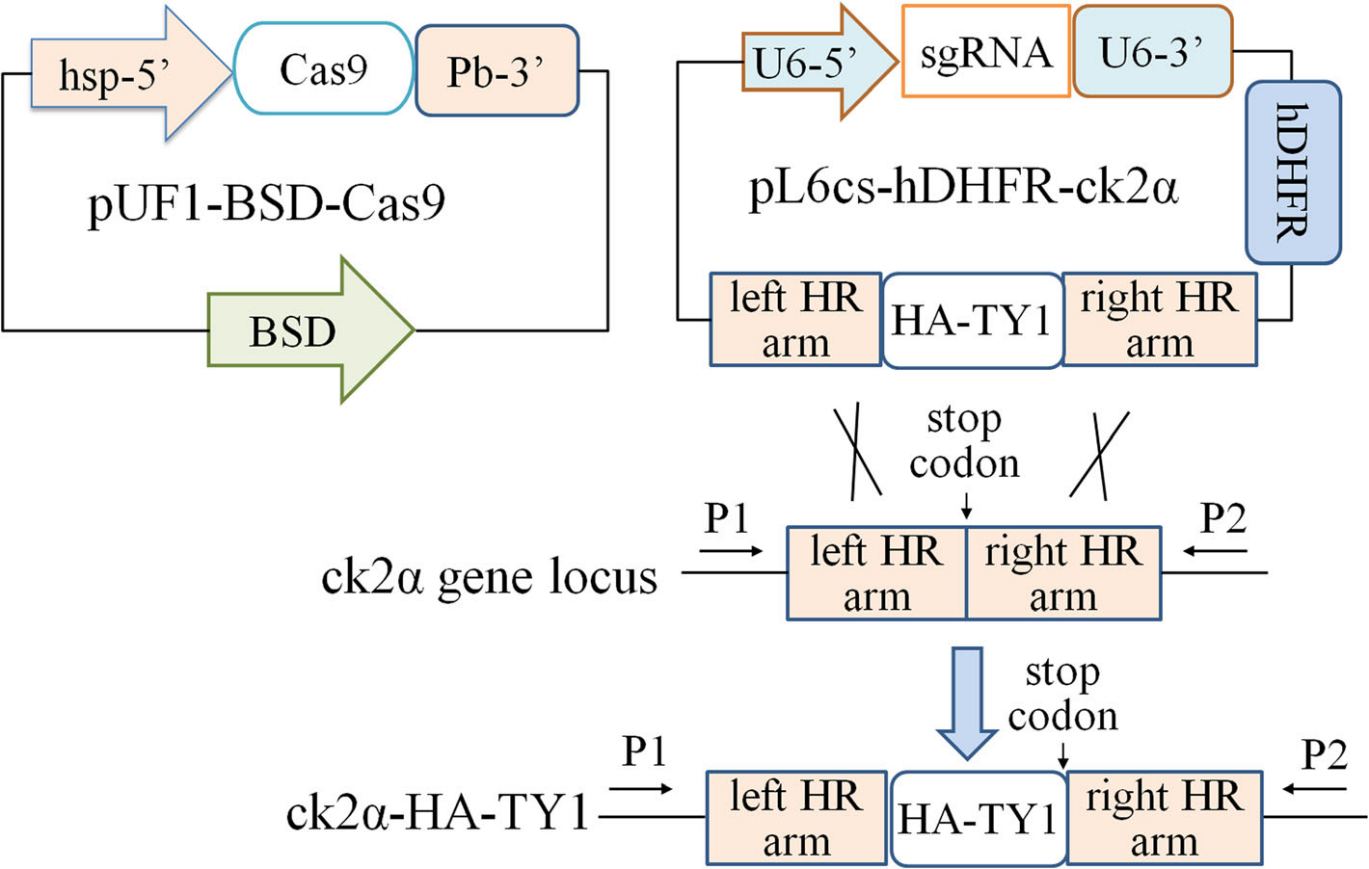
Schematic illustration of the ck2α tagging principle using CRISPR/Cas9. The HA-TY1 tag sequences are integrated into ck2α gene just before its stop codon through homologous recombination happened at left and right arms. Primers for PCR to check the tagging are labeled as P1 and P2

**Fig. 3:**
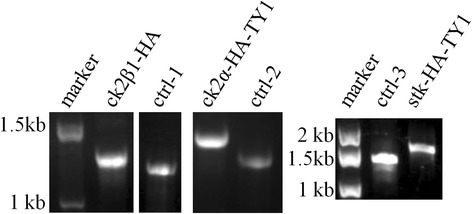
Genomic DNA PCR to confirm the tagging. Using genomic DNA as the template and specific primers of each gene (P33/P34 for ck2β1, P1/P2 for ck2α, P35/P36 for stk), the products obtained by PCR were separated on an agarose gel. The negative control was the PCR product obtained from 3D7 genomic DNA. The PCR product sizes of ctrl-1, ctrl-2 and ctrl-3, were 1175 bp, 1437 bp, and 1390 bp, respectively. After tagging, the PCR product sizes of transgenic ck2β1-HA, ck2α-HA-TY1 and stk-HA-TY1 were 1280 bp, 1650 bp, and 1603 bp, respectively

Around 20 days after electroporation, live parasites could be seen in the culture under selection with both drugs. A portion of the live parasite population was collected for genomic DNA isolation, and a PCR was performed to validate the modification of the gene of interest. In these PCRs, we always used two primers which were designed at the genomic DNA sequences beyond the left and right homologous arms (P1/P2, Fig. 2), to prevent PCR contamination from episomal plasmid template. PCR products were analyzed by agarose electrophoresis. For the three genes tested, the size of the amplification matched the expected DNA length with difference between transgenic strains and wild-type 3D7 (Fig. 3). PCR products were also sequenced to confirm that HA or HA-TY1 tags were successfully integrated into genomic DNA at the carboxy-terminal end of ck2β1, ck2α and stk. Therefore, CRISPR/Cas9 system was successfully used to add tags into endogenous genes of *P. falciparum*. To prevent the dysfunction of parasite proteins caused by tagging, the amino acid sequence G-S-G-S-G-G (G is Glycine, S is Seronine) was added as a soft linker between the tags and coding sequences of parasite genes.

### Successful tagging checked by western blot

**Fig. 4:**
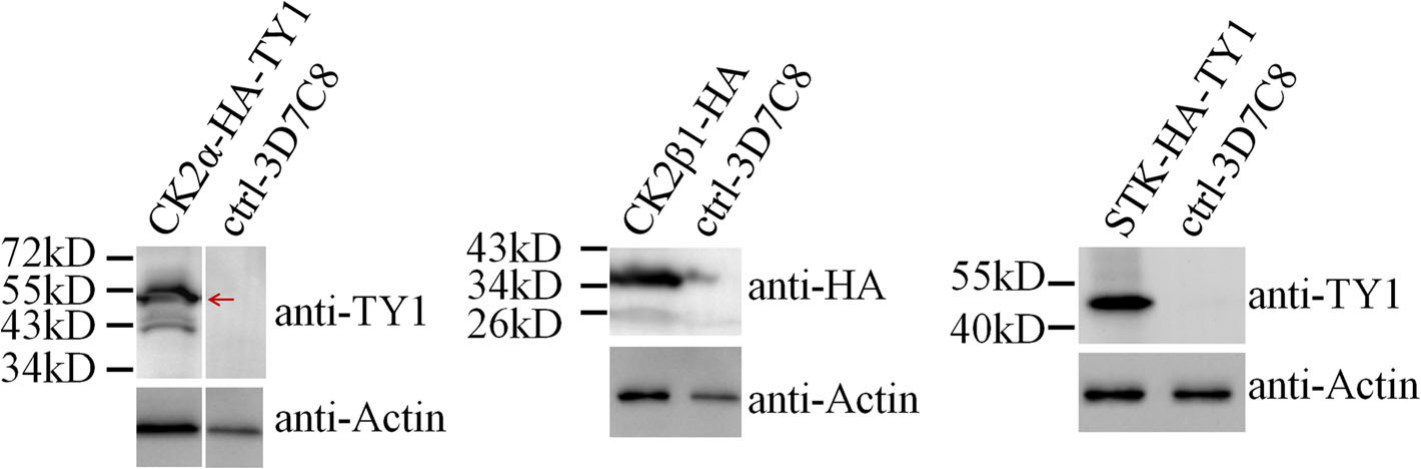
Western blot to confirm the tagging. The tags added to CK2α, CK2β1 and STK were HA-TY1, HA, HA-TY1, respectively. Therefore, mouse anti-TY1, rabbit anti-HA and mouse anti-TY1 were 1st antibodies used for the Western Blot of CK2α-HA-TY1, CK2β1-HA, STK-HA-TY1, to confirm the successful tagging. The same lysate samples were also separated on SDS-PAGE and blotted with mouse anti-Actin as the loading control. HRP-goat anti-mouse and HRP-goat anti-rabbit were the second antibodies

Transgenic parasites that had been checked by PCR and DNA sequencing were further confirmed by Western blot. Theoretical molecular weights of the proteins CK2β1-HA, CK2α-HA-TY1 and STK-HA-TY1 were 33KD, 47.7KD and 46.8KD, respectively (Fig. 4). Western blot results showed that the molecular weight of STK-HA-TY1 protein in SDS-PAGE was the same as its theoretical one. The bands corresponding to CK2β1-HA and CK2α-HA-TY1 appeared to be slightly larger than their theoretical molecular weight, which may be caused by post-translational modifications of the two proteins. Therefore, the HA or HA-TY1 tag was successfully added to the *ck2α*, *ck2β1* and *stk* genes of *P. falciparum* using CRISPR/Cas9 system. The tag was integrated into the proper open reading frames and translated with the endogenous proteins of parasites.

### Subcellular localization of STK proteins with immunofluorescence assay

**Fig. 5:**
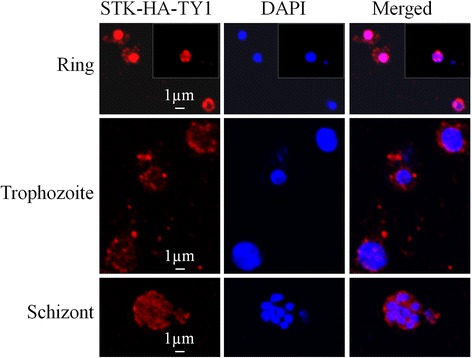
Subcellular localization of STK protein by immunofluorescence assay. Parasites at ring, trophozoite and schizont stages were collected and incubated with mouse anti-TY1 antibody and then 2nd antibody. The red fluorescence represents STK-HA-TY1; the blue fluorescence is DAPI, representing the parasite nucleus. In the images at top line, the picture highlighted by square with dashed line is IFA of a nucleus taken under other view

We used STK-HA-TY1 to analyze the subcellular localization of tagged protein. Transgenic parasites containing STK-HA-TY1 were synchronized and collected at ring, trophozoite and schizont stage. Parasites were fixed and then incubated with anti-TY1 antibody. The immunofluorescence assay (IFA) showed that STK proteins localized in the nucleus as well as at the nuclear periphery during ring stage. The localization of STK protein during trophozoite and schizont stages is similar to the distribution pattern of ring stage but with weaker signal at the nucleus (Fig. 5). This result indicated that HA-TY1 tag was successfully integrated to endogenous STK protein, and subcellular localization of STK protein could be vividly viewed using IFA. HA tag to C-terminus of Pfck2α or Pfck2β1 has been reported using episomes or old recombination method; subsequent IFAs show that these two tagged proteins localize to both cytoplasmic and nuclear compartments of parasite [26, 27].

## Discussion

The molecular study of *P. falciparum* can promote the development of malaria vaccines and anti-malarial drugs. However, only a few specific antibodies against *P. falciparum* proteins are currently available, which greatly limits molecular studies. Before the emergence of CRISPR/Cas9, the old method to edit *P. falciaprum* genes involved single or double-crossover recombination which was very inefficient and time-consuming (required several months). Recently, CRISPR/Cas9 has been used for gene editing in various organisms including *Plasmodium* [17–19]. The Cas9 endonuclease is guided to target DNA site by a sgRNA and induces DSBs at this site. Then the induced DSBs is repaired by homologous recombination using donor DNAs. In *Plasmodium*, the double-strand break repair by homologous recombination is largely favored since canonical NHEJ is deficient in this organism [20]. Up to now, the application of CRISPR/Cas9 system in *P. falciparum* was limited to gene knock out, generating genome mutation or GFP reporter parasite line with disruption of inserted sites [17, 19, 21]. A recently improved CRISPR/Cas9 system contains Cas9 nuclease, sgRNA, and a selectable marker in one plasmid while homologous arms (donor DNA fragments) without selectable marker in another plasmid. This modified system is used for DNA disruption, where promoter, 3′-UTR and GFP or other tag coding sequences are inserted to disrupt the loci instead of drug selection marker. Thus, this new system is saving one drug selection marker for consecutive gene manipulations in *P. falciparum* [28]. Efficient tagging to *Plasmodium yoelii* genes using CRISPR/Cas9 has been reported [29]. Here, we adapt the CRISPR/Cas9 system to add tags to *P. falciparum* endogenous genes. By using our adapted system, transgenic parasites could be achieved in ~20 days, significantly shorting the required time for gene tagging. Additionally, we were able to obtain three distinct tagged genes in three trials. With these tagged proteins, many molecular assays such as ChIP, IP and IFA become easy to perform.

## Conclusions

In summary, an efficient method using CRISPR/Cas9 was adapted to add tag sequences to three endogenous genes of *P. falciparum*, which provided a good example for editing parasite genes as well as laid a foundation for the molecular studies of CK2α, CK2β1 and STK proteins. This will be beneficial for our understanding of *Plasmodium* biology and consequently could improve the development of anti-malaria vaccines and drugs.

## Additional file

Additional file 1: PCR primers and sgRNAs used for plasmid construction. (PDF 78 kb)

## Abbreviations

BSD: blasticidin S deaminase
ChIP: chromatin immunoprecipitation
CK2α: casein kinase 2 alpha subunit
CK2β1: casein kinase 2 beta subunit 1
Co-IP: co-immunoprecipitation
DSB: double-strand break
HA: human influenza hemagglutinin tag
hDHFR: human dihydrofolate reducatase
IFA: immunofluorescence assay
IP: immunoprecipitation
iRBCs: infected red blood cells
sgRNA: single guide RNA
STK: a putative protein kinase which recognizes Ser/Thr motif
TY1: TY1 tag
