# Supplementary material for: Tagging to endogenous genes of Plasmodium falciparum using CRISPR/Cas9
Source: Parasit Vectors. 2017 Dec 2;10:595. doi: 10.1186/s13071-017-2539-0 (PMC5712073; doi:10.1186/s13071-017-2539-0)
Supplement: Additional file 1: — PCR primers and sgRNAs used for plasmid construction. (PDF 78 kb) [file 13071_2017_2539_MOESM1_ESM.pdf]

a. Annealed DNA for replacing yFCU expression cassette in pL6cs plasmid

5'-AGGTGACACTATAGAATACTCAAGCTGCGGCCCTAGTCTAGGGcGCGCCatgtaggcgccaact
3'-TCCACTGTGATATCTTATGAGTTCGACGCCGGGATCAGATCCCgCGCGGtacatccgcggttga

b. Primers for adding HA tag to C-terminus of ck2β1 using CRISPR/Cas9

P3 tcatctcttatcaataatagctgg
P4 ACCTCCAGAACCACTTCCgtttcagaaatttgtagttcttc
P5 CTGAcTAcGCAtaaacataaacctaaatatatttaaatttatatg
P6 aactgatcttaaaattagacatgatc
P7 acgGGAAGTGGTTCTGGAGGTTATCCTTAcGATGTTCCAGAcTATGCAATATATC
P8 atatatttaggtttatgtttaTGCgTAgTCAGGcACATC
P9 CATATTTATTAAATCTAGAATTCttatcaataatagctggggatgcac
P10 GTTATTATTTTTACCGTTccatgGgacatgatcataagaaaaaggtgtc
P11 gtattttaaaaagTTACgTaaAgaagaactacaaatttctgaaacg
P12 gttcttcTttAcGTAActtttaaaatactttggattttttttttaag
P13 CATATTAAGTATATAATATTgtattttaaaaagcttagaagttttagagctagaaatag
P14 ctatttctagctctaaaacttctaagctttttaaaatacAATATTATATACTTAATATG

c. Primers for adding HA-TY1 tag to C-terminus of ck2α using CRISPR/Cas9

P15 gcattggattattgtcatagccaag
P16 CCACTTCCtgattcTtcTCTgacttctctaaagtaaggatgctcc
P17 CCCATTAGATtaataataataaaaaaaaatgcacacatat
P18 caattgaaaatttggaaaaaaaattttatg
P19 gaagtcAGAgaAgaatcaGGAAGTGGTTCTGGAGGTTATC
P20 gtgtgcatttttttttattattattaATCTAATGGGTCTTGATTAGTGTGTAC
P21 GGCCCTAGTCTAGGGcGCGCCattggattattgtcatagccaag
P22 ATTTTTTTTACAAAATGCTTAAGgaaaaagaagacatggtttgtttgg
P23 ATATTAAGTATATAATATTgttactttagagaagtccgtggttttagagctagaaatag
P24 ctatttctagctctaaaaccacggacttctctaaagtaacAATATTATATACTTAATAT

d. Primers for adding HA-TY1 tag to C-terminus of stk using CRISPR/Cas9

P25 CCATTAGATtgatttaaaaaaaaacggaattttttttttttttaaag
P26 TTTTTTACAAAATGCTTAAGgtaagctcttcctatttcaaaatacaaatc
P27 GGCCCTAGTCTAGGGcGCGCCtttgtttttgtttgggacatatc
P28 CctcattgtcTtcTtcAaGgttaagaataatatttctgcgc
P29 acCtTgaAgaAgacaatgagGGAAGTGGTTCTGGAGGTTATC
P30 ccgtttttttttaaatcaATCTAATGGGTCTTGATTAGTGTGTAC
P31 ATATTAAGTATATAATATTgtattattcttaacttagagggttttagagctagaaatag
P32 ctatttctagctctaaaaccctctaagttaagaataatacAATATTATATACTTAATAT

e. DNA cassettes to express sgRNAs of 3 genes

ck2β1 gtattttaaaaagcttagaa**agg**(agg is the PAM)
ck2α ttactttagagaagtccgtg**agg**(agg is the PAM)
stk tattattcttaacttagagg**agg**(agg is the PAM)

f. Primers to check the tagging of 3 genes by PCR

P33 ttatatggattaaaaacggagatcc
P34 caaataaaataatatgcaaaggaatcac
P1 cctaaatttacagataaggatattcg
P2 gttgagcacatttttctctataaggg
P35 ctttgatgaagatgggaatttgtatg
P36 ccccaattctaattatatgctcaaaag
